# T1 mapping and survival in systemic light-chain amyloidosis

**DOI:** 10.1093/eurheartj/ehu444

**Published:** 2014-11-17

**Authors:** Sanjay M. Banypersad, Marianna Fontana, Viviana Maestrini, Daniel M. Sado, Gabriella Captur, Aviva Petrie, Stefan K. Piechnik, Carol J. Whelan, Anna S. Herrey, Julian D. Gillmore, Helen J. Lachmann, Ashutosh D. Wechalekar, Philip N. Hawkins, James C. Moon

**Affiliations:** 1 The Heart Hospital, 16-18 Westmoreland Street, London W1G 8PH, UK; 2 The National Amyloidosis Centre, Division of Medicine, UCL Medical School, Royal Free Hospital, Rowland Hill Street, London NW3 2PF, UK; 3 Institute of Cardiovascular Science, University College London, Gower Street, London WC1E 6BT, UK; 4 Biostatistics Unit, UCL Eastman Dental Institute, 256 Grays Inn Road, London WC1X 8LD, UK; 5 Oxford Centre for Clinical Magnetic Resonance Research, Division of Cardiovascular Medicine, Radcliffe Department of Medicine, University of Oxford, Oxford OX3 9DU, UK

**Keywords:** ECV, Amyloid, CMR, Cardiomyopathy, Heart failure, T1 mapping

## Abstract

**Aims:**

To assess the prognostic value of myocardial pre-contrast T1 and extracellular volume (ECV) in systemic amyloid light-chain (AL) amyloidosis using cardiovascular magnetic resonance (CMR) T1 mapping.

**Methods and results:**

One hundred patients underwent CMR and T1 mapping pre- and post-contrast. Myocardial ECV was calculated at contrast equilibrium (ECV_i_) and 15 min post-bolus (ECV_b_). Fifty-four healthy volunteers served as controls. Patients were followed up for a median duration of 23 months and survival analyses were performed. Mean ECV_i_ was raised in amyloid (0.44 ± 0.12) as was ECV_b_ (mean 0.44 ± 0.12) compared with healthy volunteers (0.25 ± 0.02), *P* < 0.001. Native pre-contrast T1 was raised in amyloid (mean 1080 ± 87 ms vs. 954 ± 34 ms, *P* < 0.001). All three correlated with pre-test probability of cardiac involvement, cardiac biomarkers, and systolic and diastolic dysfunction. During follow-up, 25 deaths occurred. An ECV_i_ of >0.45 carried a hazard ratio (HR) for death of 3.84 [95% confidence interval (CI): 1.53–9.61], *P* = 0.004 and pre-contrast T1 of >1044 ms = HR 5.39 (95% CI: 1.24–23.4), *P* = 0.02. Extracellular volume after primed infusion and ECV_b_ performed similarly. Isolated post-contrast T1 was non-predictive. In Cox regression models, ECV_i_ was independently predictive of mortality (HR = 4.41, 95% CI: 1.35–14.4) after adjusting for E:E′, ejection fraction, diastolic dysfunction grade, and NT-proBNP.

**Conclusion:**

Myocardial ECV (bolus or infusion technique) and pre-contrast T1 are biomarkers for cardiac AL amyloid and they predict mortality in systemic amyloidosis.

**See page 203 for the editorial comment on this article (doi:10.1093/eurheartj/ehu442)**

## Introduction

Systemic amyloid light-chain (AL) amyloidosis is a multiorgan, infiltrative disorder caused by an underlying plasma cell dyscrasia and is characterized by tissue and organ amyloid deposition with interstitial expansion. Cardiac involvement is present in ∼50% of patients at presentation and is the principal driver of prognosis. Treatment comprises chemotherapy or autologous stem cell transplantation to suppress clonal light-chain production, which may retard disease progression or facilitate regression.^1^

Current predictors of survival rely on measuring surrogate rather than direct markers of interstitial expansion. Concentration of the serum biomarkers NT-proBNP and Troponin T form the basis of the Mayo Staging classification^2^ but are influenced by renal impairment which is present in a quarter of patients at presentation. ECG criteria, low limb lead voltages,^3^ or fragmented QRS complexes^4^ are also predictive, but are confounded by pericardial effusions and conduction abnormalities. Echocardiographic parameters also predict outcome,^5–7^ but coexisting causes of left ventricular hypertrophy or diastolic impairment may affect interpretation.

Cardiovascular magnetic resonance (CMR) using the late-gadolinium enhancement (LGE) technique adds value in the diagnosis of cardiac involvement in AL amyloidosis. Altered gadolinium kinetics also shows some correlation with survival.^8^ Recently we have shown that pre-contrast, native myocardial T1 mapping correlates with cardiac disease burden and detects early disease.^9^ T1 mapping pre- and post-contrast can be used to derive the partition coefficient and, with the haematocrit, the myocardial extracellular volume (ECV)^10–12^ which is a direct measurement of myocardial interstitium and therefore likely a surrogate marker of amyloid burden.^13,14^ The ECV can also assess amyloid burden in other organs.^15^ Furthermore, in other cardiac diseases, myocardial ECV predicts outcome.^16^

Technically, measurement of the ECV requires equilibration of contrast concentrations between blood and myocardium, which can be achieved precisely using a somewhat cumbersome primed contrast infusion,^10^ or sufficiently through delayed study following administration of a bolus of gadolinium. Both techniques measure ECV the same where the ECVs are typically 0.4 or less, but one paper has shown a bias towards over-estimation of the true ECV in high ECV conditions (including *n* = 20 amyloid patients).^17^

We hypothesized firstly that the myocardial ECV and pre-contrast T1 would correlate with disease burden in cardiac AL amyloidosis as assessed by current measures. Additionally, we tested the ability of both biomarkers as predictors of survival in AL amyloidosis by comparing the predictive power of: ECV after primed infusion (ECV_i_); pre-contrast T1; bolus-only ECV (ECV_b_); and post-contrast T1.

## Methods

The research was approved by The UCL/UCLH Joint Committees on the Ethics of Human Research Committee and all participants provided informed, written consent prior to enrolment. One hundred consecutive patients with systemic AL amyloidosis who were assessed between 2010 and 2012 at the National Amyloidosis Centre (Royal Free Hospital, London, UK) and in whom there were no contraindications to CMR (presence of non-MR compatible devices) or contrast administration (GFR < 30 mLs/min) or potential confounders to T1 measurement (known atrial fibrillation at first visit) were recruited. These 100 patients include all 60 patients studied previously in the baseline study.^13^ Approximately 25% of patients with systemic AL amyloidosis seen at the centre during this period had an eGFR of <30 mL/min/1.73 m^2^ and were therefore excluded. Six patients who were found to have atrial fibrillation/flutter once in the scanner after they had consented were not excluded.

All patients had histological proof of systemic AL amyloidosis except 2 (2%), who died before biopsy could be undertaken, but in whom monoclonal gammopathies were present and the organ distribution of amyloid on SAP scintigraphy was characteristic of AL type. Histology was performed with Congo red followed by immunohistochemical staining; tissues examined were: kidney (26%), endomyocardium (7%), bone marrow (13%), upper gastrointestinal tract (7%), liver (3%), fat (15%), spleen (1%), lung (1%), rectum (9%), soft tissues (12%, included skin, tongue, buccal mucosa, labia), lymph node (3%), and peritoneum (1%).

All patients underwent 12 lead ECG, assays of the cardiac biomarkers NT-proBNP and Troponin T, and echocardiography at baseline. Mean ECG QRS voltage in limb and praecordial leads were calculated.^18^ Echocardiographic assessment of diastolic function was performed using the E:E′ ratio. Where transmitral E-wave deceleration time and isovolumetric relaxation time were available, a diastolic dysfunction grade of 0–3 was assigned according to established British Society of Echocardiography (BSE) criteria.

All additionally underwent conventional CMR on a 1.5 T magnet (Avanto, Siemens). T1 mapping was performed using the Shortened Modified Look Lockers Inversion (ShMOLLI) recovery sequence^19^ pre- and post-contrast (0.1 mmol/kg bolus and 0.0011 mmol/kg/min infusion of Dotarem™) as part of the Equilibrium CMR (EQ-CMR) technique, the post-contrast T1 map being performed at 15 min and after equilibration (mean time from bolus 45 min), as previously described.^10^

### Analysis

**Figure 1:**
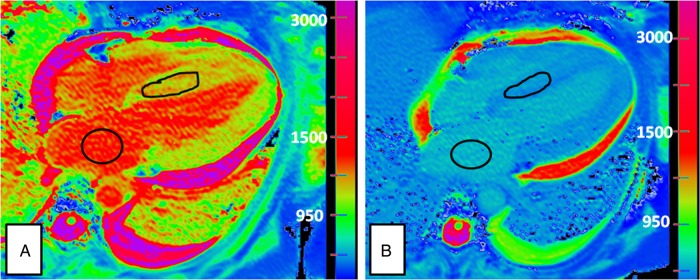
Showing (*A*) pre-contrast and (*B*) post-contrast, four-chamber ShMOLLI image with regions of interest drawn in the left atrium for blood T1 measurement and in the basal septum of left ventricle, excluding at least the first two pixels of endocardium on either side of the septum in order to avoid through planing of blood pool.

Standard CMR parameters of structure (left ventricle (LV) mass, left atrial area with/without indexing for body surface area, maximal septal thickness) and systolic function [ejection fraction, mitral annular plane systolic excursion (MAPSE), Tricuspid annular plane systolic excursion (TAPSE)] were assessed. A region of interest (ROI) was drawn in the basal septum in a four-chamber view in all patients and in the left atrium for blood T1 measurement as papillary muscle hypertrophy made drawing an ROI in the LV cavity challenging (see *Figure 1*); ROIs were mid-myocardial (at least two pixels away from the apparent blood:myocardial boundary) and were drawn without reference to the LGE images (see *Figure 1*).^13^ We quantified interstitial expansion with the ECV as described previously: ECV = *λ*(1 − haematocrit), where *λ* = [ΔR1_myocardium_]/[ΔR1_blood pool_] pre- and post-Gd (where R1 = 1/T1).

Some ECV data (*n* = 19 of the 100) pre-dated availability of ShMOLLI T1 mapping and had utilized multibreath-hold T1 measurement.^10^ We have demonstrated equivalence of ECV values derived from this technique with ShMOLLI ECV and so these data were not excluded from the analyses.^20^

That said, these patients did not have an ECV_b_ value or the subsidiary component of the ECV equation, pre-contrast T1; multibreath-hold measurement has been shown to be inferior to T1 mapping, so accordingly, these were excluded from the sub-analysis comparing techniques—this particular analysis therefore consists of 81 rather than 100 patients.

Extracellular volume and myocardial T1 results were compared with 54 healthy volunteers who underwent pre-contrast T1 mapping and ECV measurement (bolus and infusion). The number of patients dead and alive was assessed after a median duration of 23 (interquartile range: 6–25) months. Some analysis involved sub-grouping patients into pre-test probability of cardiac involvement. This was done as previously described and as stated below.

Definite cardiac involvement—any of:

- Left ventricle (LV) wall thickness of ≥12 mm by echocardiography in the absence of any other known cause
- Right ventricle (RV) free wall thickening co-existing with LV thickening by echocardiography in the absence of systemic or pulmonary hypertension

Possible cardiac involvement—any of:

- LV wall thickening by echocardiography in the presence of hypertension
- RV thickening by echocardiography in the presence of pulmonary hypertension
- Normal wall thickness by echocardiography with diastolic dysfunction and raised serum biomarkers^2^

No suspected involvement:

- Normal wall thickness by echocardiography with normal serum biomarkers

### Statistical analysis

Study data were collected and managed using REDCap (Research Electronic Data Capture) electronic data capture tools hosted at University College London.^21^ Analysis was performed using SPSS (IBM Corp. Released 2012. IBM SPSS Statistics for Windows, Version 21.0. Armonk, NY: IBM Corp), R programming language for statistical computing (version 3.0.1, The R Foundation for Statistical Computing) and in Stata (StataCorp. 2011. Stata Statistical Software: Release 12. College Station, TX: StataCorp LP). All continuous variables were normally distributed except NT-proBNP and Troponin T which were log (ln) transformed (base e) to achieve normality for further analysis. Linear regression models measured the association between quantitative ECV and other variables; variance inflation factors <2 excluded collinearity.

Pearson's correlation coefficients are presented in terms of *R* values. Means are presented ±SD. The *χ*^2^ test was used to compare categorical variables between patients and controls whilst the unpaired *t*-test was used to compare continuous variables between the patients and controls. A one-way ANOVA with Bonferroni correction was used to test ECV with pre-test clinical probability of cardiac involvement.^13^

To test the prognostic value of ECV and pre-contrast T1, survival was evaluated using Cox proportional hazards regression analysis, providing estimated hazard ratios (HRs) with 95% confidence intervals (CIs) and Kaplan–Meier curves. Conventional ROC analysis could not be performed because the follow-up period was not the same for each patient. Therefore, time-dependent ROC curves^22^ were used to assess the capacity of ECV_i_ compared with ECV_b_ and pre-contrast myocardial T1 for discriminating between surviving and dying patients with AL amyloidosis. For fixed times (*t* = 12 months, *t* = 24 months) and specificity level, we have compared the sensitivity of ECV_i_, ECV_b_, and pre-contrast myocardial T1 measurements for detecting patients who will die by time *t*. For the ROC curves constructed using the nearest neighbour estimator (NNE) we used a narrow span of *λ* (0.25 × nobs^−0.20^) to yield only moderate smoothing. To permit comparison of ROCs by the NNE estimator, a set of simple KM estimator ROC curves for this data at *t* = 24 months are also provided (see Supplementary material online, *Figure S1*).

Optimal myocardial T1 and ECV values were explored by Cox regression, using the median and the 1st or 2nd tertiles as cut-off values. The two groups resulting from each cut-off were compared using the Harrell's C statistic (a measure of discrimination between groups) to determine the better model and thus biomarker for predicting survival. All variables were first explored with univariate Cox regression. Multivariable models evaluated the independent predictive value of ECV above other clinically and statistically significant covariates.

## Results

*Table 1* summarizes baseline characteristics for patients and healthy volunteers. Within the patient cohort, 14 (14%) patients were on treatment for hypertension; 10 (10%) had confirmed coronary artery disease by angiography, 1 (1%) had had a stroke, and 2 (2%) had diabetes. Fifty patients were treated with chemotherapy for the first time which comprised triple therapy with either cyclophosphamide, thalidomide, and dexamethasone or cyclophosphamide, bortezomib and dexamethasone (CVD), depending on local guidelines of regional NHS Trusts within the UK. Seventeen patients were treated for a 2nd or 3rd time having relapsed—treatment was either with CVD or a lenalidomide-containing regimen in these instances. Nine patients had not received any chemotherapy as there was no clinical indication (e.g. renal amyloid with established renal failure, isolated neuropathic presentations) and 24 patients were under a stable follow-up with no indication for further chemotherapy at the time of scan.

**Table 1:** Baseline characteristics of the 100 AL amyloidosis patients and 54 healthy controls

Characteristic	Patients	Healthy controls	*P* value
Male/female	67/33	25/29	0.01
Mean age ± SD (years)	62 ± 10	46 ± 15	0.001
Mean creatinine ± SD (mmol/L)	89 ± 32	74 ± 13	0.001
Mean NYHA (I/II/III/IV)	29/56/15/0	–	
Mean EF ± SD (%)	66 ± 11	67 ± 6	0.42
Diastolic dysfunction grade (0/1/2/3)^a^	15/36/25/22	–	
Mean indexed end-diastolic LV volume ± SD (mLs)	60 ± 14	73 ± 12	0.001
Mean indexed end-systolic LV volume ± SD (mLs)	19 ± 10	25 ± 7	0.001
Mean indexed LV mass ± SD(g/m^2^)	96 ± 34	65 ± 15	0.001
Mean indexed LA area	13 ± 3	9 ± 1.5	0.001
Median NT-proBNP in pmol/L (IQ range)	146 (38–359)	–	
Median troponin T in ng/L (IQ range)	0.03 (0.01–0.06)	–	
AF/atrial flutter (%)	6 (6)	0	

^a^Two patients did not have all three diastolic markers measured due to poor windows and therefore could not be graded as per BSE guidelines.

Twenty-one patients had a pre-test probability of no cardiac involvement, 26 had possible cardiac involvement, and 53 had definite cardiac involvement.

All ECV values are the ECV_i_ from infusion measurement unless otherwise stated. Healthy controls were younger on average, but our work and others suggests any ECV changes with age are small compared with amyloid changes.^23,24^ There were proportionately more females in the control vs. the patient group. This slightly increases the control group ECV and pre-contrast T1 compared with that of a gender-matched group (male vs. female: ECV 0.24 vs. 0.27, *P* < 0.001; T1 940 vs. 966 ms, *P* = 0.006).

As in previous work, mean cardiac ECV was greater in patients compared with healthy volunteers with a wider range (0.44 ± 0.12 vs. 0.25 ± 0.02, *P* < 0.001) and correlated with pre-test probability of cardiac involvement by conventional parameters (*P* < 0.001)^13^ (see Supplementary material online, *Figure S2*). Mean pre-contrast myocardial T1 values were raised in patients compared with healthy volunteers (1080 ± 87 ms vs. 954 ± 34 ms, *P* < 0.001) and also correlated with pre-test probability of cardiac involvement (*P* < 0.001).^9^ *Table 2* provides the Pearson correlation coefficients of ECV, pre-contrast and post-contrast myocardial T1 to other cardiac parameters, many of which typically change in cardiac amyloid. Extracellular volume correlated significantly with 17 of 19; pre-contrast T1 with 12, and post-contrast T1 with 10.

**Table 2:** Extracellular volume, pre-, and post-contrast T1 correlations (Pearson's *R* correlations) with cardiac structure and function, biomarkers and ECG changes in light-chain amyloid patients

	ECV		Myocardial T1			
			Pre-contrast		Post-contrast	
	*R*	*P*-value	*R*	*P*-value	*R*	*P*-value
LV structure by CMR						
LV mass	0.49	<0.001	0.44	0.001	0.44	0.001
Indexed LV mass	0.50	0.001	0.44	0.001	0.41	0.001
Septal thickness	0.61	0.001	0.54	0.001	0.35	0.001
LA area	0.31	0.002	0.13	0.25	0.22	0.054
Indexed LA area	0.29	0.003	0.11	0.35	0.11	0.35
LV systolic function by CMR						
Ejection fraction	0.46	0.001	0.31	0.004	0.22	0.045
MAPSE^^a^^	0.59	0.001	0.53	0.001	0.25	0.023
LV end-diastolic volume	0.05	0.64	0.12	0.28	0.15	0.18
LV end-systolic volume	0.31	0.002	0.15	0.19	0.06	0.58
Indexed LV end-diastolic volume	0.10	0.32	0.17	0.13	0.27	0.01
Indexed LV end-systolic volume	0.31	0.002	0.13	0.23	0.04	0.73
LV diastolic function by echo						
E:E′	0.35	0.001	0.25	0.03	0.34	0.002
IVRT	0.37	0.002	0.32	0.02	0.19	0.17
E-deceleration time	0.24	0.02	0.37	0.001	0.07	0.51
RV systolic function by CMR						
TAPSE^$^b^^	0.53	0.001	0.50	0.001	0.42	0.001
Biomarkers						
Serum NT-pro-BNP (LnNT-proBNP)	0.65	0.001	0.58	0.001	0.35	0.001
Troponin T (lnTropT)	0.43	0.02	0.28	0.07	0.24	0.12
ECG						
ECG limb lead mean voltage	0.43	0.001	0.37	0.001	0.10	0.40
ECG chest lead mean voltage	0.22	0.03	0.13	0.27	0.24	0.03

^a^Mitral annular plane systolic excursion.

^$b^Tricuspid annular plane systolic excursion.

**Figure 2:**
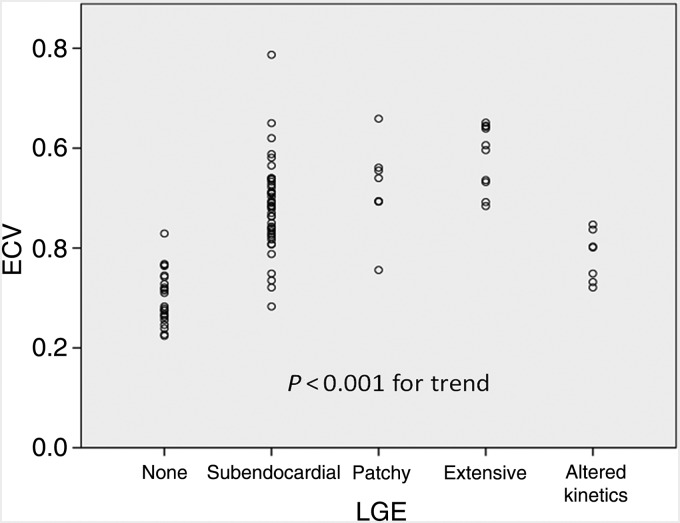
Dot plot showing correlation between extracellular volume and late-gadolinium enhancement.

With regards LGE, 25 patients had no LGE, 50 had global subendocardial enhancement, and 10 had extensive enhancement. Eight had patchy enhancement and seven had evidence of only altered gadolinium kinetics, i.e. reversed nulling of myocardium and blood after gadolinium administration. Extracellular volume correlated significantly with increasing degrees of LGE (*P* < 0.001) as shown in *Figure 2*.

At follow-up (median 23 months), 25 of 100 patients had died. For each potential predictor, median and tertile cut-points were assessed for predictive power and the best result presented (see *Table 3*).

**Table 3:** Median and tertiles for extracellular volume after primed infusion and pre-contrast T1 with associated Hazard Ratios by Cox regression and Harrell's *C* statistic

	Tertile	Cut-point	HR (95% CI)	*P*-value	Harrell's *C* statistic
ECV_i_	Tertile 1	0.40	4.67 (1.39–15.5)	0.013	0.63
	Median	0.45	3.83 (1.53–9.61)	0.004	0.66
	Tertile 2	0.49	3.61 (1.56–8.38)	0.003	0.65
Pre-contrast myocardial T1	Tertile 1	1044 ms	5.39 (1.24–23.4)	0.02	0.64
	Median	1080 ms	3.01 (1.08–8.44)	0.035	0.62
	Tertile 2	1116 ms	3.22 (1.30–8.04)	0.01	0.63
ECV_b_	Median	0.44	5.09 (1.09–23.7)	0.03	0.68
Post-contrast T1	Median	565 ms	0.45 (0.17–1.20)	0.11	0.41

Cox regression and Harrell's *C* statistic also shown for median ECV_b_ and post-contrast T1.

**Figure 3:**
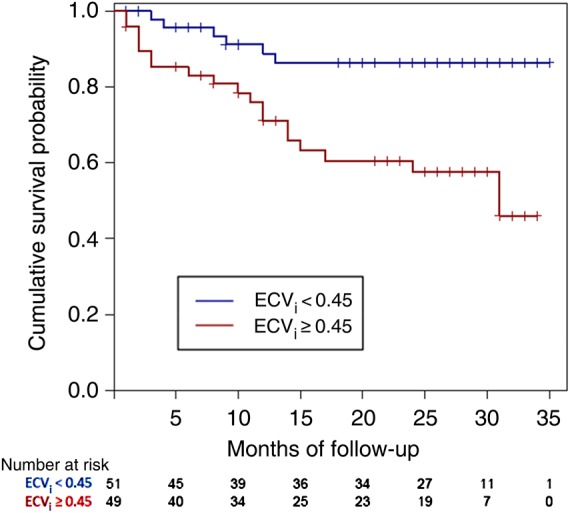
Kaplan–Meier survival curves for extracellular volume after primed infusion.

**Figure 4:**
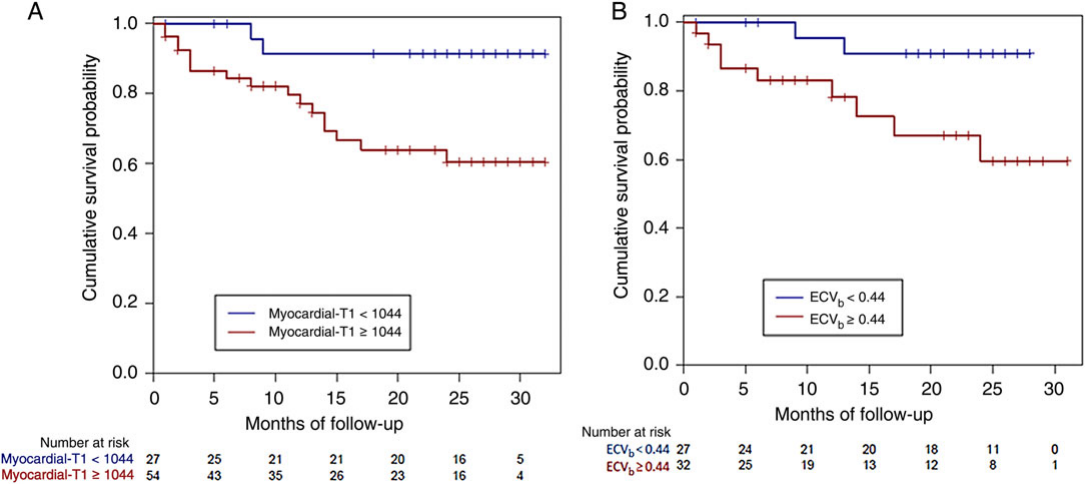
Kaplan–Meier survival curves for (*A*) pre-contrast myocardial T1 and (*B*) extracellular volume at bolus (NB: although the median of 0.44 was used, the groups are not equal because more than 1 patient had an extracellular volume of 0.44).

For ECV_i_, a median ECV of 0.45 was the best predictor of survival: HR 3.84 (1.53–9.61), *P* = 0.004 (*Figure 3*). The survival curve indicates that there is an ∼40% chance of death at 23 months in patients with an ECV ≥ 0.45 compared with 15% for patients with an ECV < 0.45. For pre-contrast myocardial T1, the 1st tertile (cut-point 1044 ms) was the best predictor: HR 5.39 (1.24–23.4), *P* = 0.02 (*Figure 4A*). ECV_b_ with median of 0.44 also predicted survival with an HR of 5.09 (1.09–23.7), *P* = 0.04 (*Figure 4B*). Post-contrast T1 did not predict survival (HR = 0.5, *P* = 0.11).

**Figure 5:**
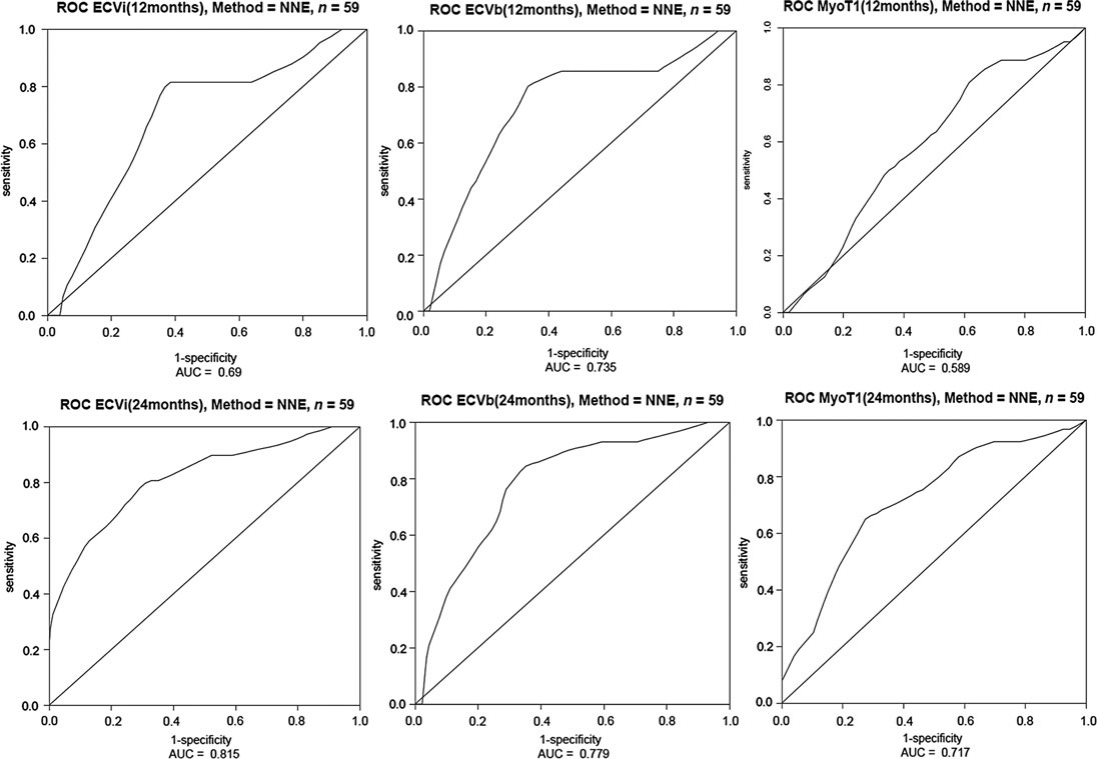
Time-dependent receiver operating characteristic curves for extracellular volume after primed infusion, extracellular volume at bolus and pre-contrast myocardial T1 and survival using nearest neighbour estimator method at time: (top 3) 12 months and (bottom 3) 24 months.

When the three predictive models ECV_i_, ECV_b_, and pre-contrast T1 were compared to determine the stronger discriminator using the Harrell's C statistic (the higher the number, the stronger discriminator), all three performed similarly (see *Table 3*). The time-dependent ROC analysis revealed that overall (considering both earlier [*t* = 12] and later [*t* = 24] follow-up times), the three ROC curves for ECV_i_, ECV_b_, and pre-contrast T1 show quite similar discrimination for cumulative mortality (see *Figure 5*).

The value ECV_i_ > 0.45 remained significantly associated with mortality (HR = 4.41, 95% CI 1.35–14.4; *P* = 0.01) in multivariable Cox models that included measures of systolic and diastolic function and serum biomarkers: E:E′, diastolic dysfunction grade (≥2), ejection fraction, and LnNT-proBNP (troponin was not available in all patients). E:E′ and NT-ProBNP also remained independently predictive.

## Discussion

Extracellular volume is the first, non-invasive quantifier of the cardiac interstitium, while pre-contrast T1 is a composite measure of interstitium and myocardial cells. This study demonstrated that ECV and native myocardial T1 as measured by the newer T1 mapping techniques, both correlate with current markers of disease severity in cardiac AL amyloidosis, supporting previous work.^11–13^ We have also demonstrated that both these biomarkers have ‘real-world’ clinical significance in that both are predictors of mortality in AL amyloidosis.

Amyloidosis is the exemplary interstitial disease of the myocardium. Cardiac involvement portends a poor prognosis which has driven the need for better methods of detecting early cardiac disease. We previously described both pre-contrast T1 mapping and ECV measurement as potential, non-invasive techniques for directly measuring the cardiac AL disease burden in amyloid.^13^ Here, these early results are strengthened by increased numbers and, additionally, prognostic significance of the biomarkers is demonstrated, even with therapy. The half of AL amyloid patients with an ECV > 0.45 had a three- to four-fold increased likelihood of death—roughly a 35–40% chance of death at 23 months compared with lower ECV patients despite therapy, lending support to ECV as a key amyloid biomarker.

Recent work published in this journal in non-amyloid patients, where ECV likely measures diffuse fibrosis, has also showed predictive power—in 1176 consecutive CMR referral patients over a median of 1.3 years follow-up, 24 deaths occurred with ECV carrying a hazard ratio of 1.52 (1.21–1.89) for admissions with heart failure and all-cause mortality.^16^ Here, we have demonstrated that ECV adds incremental value over and above existing clinical markers when risk-stratifying patients. Unfortunately, it was not possible to include NYHA class in the multivariable model because this information was not available in all patients due to other factors limiting exertion such as peripheral and autonomic neuropathy due to systemic amyloidosis. Additionally, the limited number of deaths limits very extensive multivariable analysis so this may not represent the optimal multivariable model.

We used an arbitrary categorization for the presence or otherwise of cardiac amyloid. The Mayo staging system is the most recognized predictor of survival in systemic AL amyloidosis.^2^ In new presentations, median survival was reduced from 26 to 10 months when either NT-proBNP or Troponin T was raised and reduced further still to only 3 months if both biomarkers were raised, although the authors are in the process of further refining this model with inclusion of values for serum-free light-chain concentration.^25^ Our survival data in the 49 patients scanned at presentation is currently underpowered to determine any incremental benefit of ECV in this specific patient group; this remains work in progress.

Extracellular volume is predictive, regardless of treatment status and indeed irrespective of whether patients are presenting at diagnosis or years into the disease process. Some patients in the cohort had modest ECV increases (ECV 0.30–0.40) without any other evidence of cardiac involvement (no LGE, no wall thickness increase, and no biomarker elevation), reinforcing our original findings that even patients classified as having no cardiac amyloid do in fact have raised ECVs, suggestive of low-grade cardiac disease. A plausible role for aggressive therapy in such patients to prevent progression to overt cardiac disease can be entertained.^13^

Although T1 mapping is now more mature with sequences available on all platforms, it is difficult to compare ECV and T1 to LGE because, as previously stated, the absence of LGE likely does not equate to the absolute absence of cardiac amyloid and PSIR imaging which can be helpful with LGE imaging in amyloid, is not available on all platforms. We have previously demonstrated that when ROIs are drawn in LGE-positive and LGE-negative areas in the same patient, the ECV, whilst lower in LGE-negative areas, is still not normal.^13^

The simpler pre-contrast myocardial T1 technique does not require a contrast agent and shows promise,^9^ particularly as 20–30% of patients with systemic AL amyloidosis have an eGFR of <30 mL/min at presentation and in these patients, the Mayo staging system is in part confounded by elevation of serum biomarkers due to renal dysfunction. Here, pre-contrast myocardial T1 by ShMOLLI is an alternative to ECV. It is an equally strong predictor but as mentioned earlier, it represents a composite signal from cells and interstitium, not just the interstitium alone like ECV. Some work may be needed to derive normal pre-contrast T1 values in patients with renal impairment due to non-amyloid-related pathologies. An additional issue is that pre-contrast T1 presents greater standardization challenges.

From a practical perspective, the bolus only approach to ECV (ECV_b_) was as good as ECV_i_. Our previous work showed that, in most disease states, ECV_b_ carried excellent agreement with ECV_i_ when the tissue ECV was <0.4, but generated higher results in high ECV scenarios—such as areas of scar and amyloid.^17^ Nonetheless, this study suggests that the ECV_b_ passes a key clinical utility test of being prognostic. Post-contrast T1, however, was not useful either at baseline or to predict outcome.

Limitations of the study are that patients were followed up for different time periods and are at different disease and treatment stages, with treatment here reflecting current UK practice. The causes of death are not known as patients die locally and the National Amyloidosis centre receives only notification of death rather than cause of death; however, it is widely accepted that most deaths are cardiac. Studies looking to correlate ECV change with haematological and clinical response as well as histology in AL amyloidosis have yet to be performed. Whole heart ECV calculations were not possible in this study because of through-planning of blood pool (due to cardiac motion in the superior–inferior plane) in areas of thinner myocardium towards the apex but as technology advances with motion correction T1 mapping sequences, this will become possible. As stated earlier, the number of events limits extensive analysis. That said, these are nevertheless hard endpoints and multivariable analysis can still be performed in these situations.^26^ Whether ECV and pre-contrast T1—which are not entirely concordant—provide different pathological insights is at this stage unknown.

## Conclusions

The myocardialextracellular volume, ECV, is a recently developed, non-invasive quantifier of cardiac amyloidosis. We confirm earlier results that ECV increases with established disease and detects early cardiac involvement. Extracellular volume after primed infusion is a promising clinical biomarker which passes a key test as a predictor of mortality. The pre-contrast T1 mapping method and the faster bolus-only ECV measurement techniques are equally prognostic, providing options for patients in renal failure or, in combination add robustness and diagnostic confidence.

## Supplementary material

Supplementary material is available at *European Heart Journal* online.

## Funding

This work was supported by GlaxoSmithKline for some of our research studies in amyloid and are supported by researchers at the National Institute for Health Research University College London Hospitals Biomedical Research Centre. Funding to pay the Open Access publication charges for this article was provided by the British Heart Foundation.

**Conflict of interest:** S.M.B. reports the following disclosure: Project part funded by GSK. A.D.W. reports the following disclosure: received money in relation to consultancy with GSK. J.C.M. reports the following disclosure: consultancy agreement with GSK subsequent to this manuscript being submitted. S.K.P. reports the following disclosure: NIHR BRC Research grant. All other authors do not have anything to disclose.
